# Establishing a prediction model of infection during the intravesical instillation of bladder cancer: a multicenter retrospective study

**DOI:** 10.7150/jca.45055

**Published:** 2020-04-27

**Authors:** Song Chen, Yun Yang, Ziyi Luo, Haiqing Deng, Tiancheng Peng, Zhongqiang Guo

**Affiliations:** 1 Department of Urology, Zhongnan Hospital of Wuhan University, Wuhan 430071, China; 2 Department of Dermatology, The First Affiliated Hospital of Harbin Medical University, Harbin 150001, China; 3 Department of Hematology, Zhongnan Hospital of Wuhan University, Wuhan 430071, China; 4 Department of Urology, Xiangyang Central Hospital, Xiangyang 441021, China

**Keywords:** Bladder cancer, TURBT, Intravesical instillation, Infection, Prediction model

## Abstract

**Objective**: To explore the independent risk factors of infection during the intravesical instillation of bladder cancer and establish a prediction model, which may reduce probability of infection for bladder cancer patients.

**Material and Methods**: 533 patients with newly discovered NMIBC at two hospitals from January 2017 to December 2019 were enrolled in this study. The patients were divided into “infection positive group” and “infection negative group”. The clinical data of the two groups were analyzed by logistic regression analyses. Nomogram was generated and ROC curve, calibration curve were structured to assess the accuracy of nomogram. An independent cohort included 174 patients from another hospital validated the nomogram prediction model.

**Results**: Of 533 patients, 185 patients had an infection. Univariate and multivariate logistic regression analyses showed diabetes mellitus, hemiplegia, patients without antibiotics and perfusion frequency (≥2 times/month) were the independent risk factors. AUC of the ROC was 0.858 (0.762-0.904). The nomogram could predict the probability of infection during the intravesical instillation of bladder tumor calibration curve showed good agreement. The external data validation gained good sensitivity and specificity, which indicated that the nomogram had great value of prediction.

**Conclusions**: Individualized prediction of the probability of infection may reduce the incidence of infection by argeted preventive measures.

## Introduction

Bladder cancer (BCa) belongs to a high-risk tumor in urinary malignant tumor which kills 165,000 people every year in the world 1-2. More than 70% of these patients are preliminary diagnosis diagnosed with non-muscle invasive bladder cancer (NMIBC) 3, but 50-70% of them will recur after treatment and 10-20% will progress into muscle-invasive bladder cancer (MIBC) 1. The prognosis will be worse once NMIBC progresses into MIBC.

Transurethral resection of bladder tumor (TURBT) is the first-line therapy for NMIBC 4. As the most common treatment of NMIBC, TURBT has the advantages of less trauma, less bleeding and quick recovery after surgery 5-6. However, TURBT is a surgical operation performed in the urethra, and it is inevitable that there is a risk of infection 7-8. Moreover, long-term intravesical instillation is needed after TURBT for reducing the recurrence and progress risks 9, which is likely to cause infection 10-11. Some studies showed that infection rate after urological surgery was 8.8%, which was on the top 10 of hospital infection, especially in intravesical instillation 12-13. Hydroxycamptothecin, Epirubicin, pyirubicin and gemcitabine are used for perfusion. Long term perfusion usually cause infection and affect life quality of patients, even more serious complications 14. In addition, the perfusion drug with BCG might induce systemic side effect, such as fever or skin reaction. Some patients who have underwent intravesical instillation for a long time developed anxiety 15. Previous studies have reported more than 70% cases have met with infection during the intravesical instillation therapy, a few of them develop severe infection and even septic shock 16-17. It not only increased physical pain and economic burden, but also easily exacerbated the resistance rsychology of patients to intravesical instillation. The infection prevention has great significance for improving the patient's ability of adherence and accelerating recovery.

In this study, we conducted a multicenter retrospective study to explore the influencing and independent risk factors of infection during the intravesical instillation of bladder cancer. Moreover, we established a nomogram model to predict the infection of the intravesical instillation.

## Materials and Methods

### Study patients

A development cohort and a validation cohort were included in this research. The development cohort contained 533 cases with newly diagnosed NMIBC (Ta-T1) at Zhongnan Hospital of Wuhan University (396 patients) and Renmin Hospital of Wuhan University (137 patients) from January 2017 to December 2019 were enrolled in this study.

Transurethral resection of bladder tumor (TURBT) was performed in all patients. The clinical tumor stage, tumor grade and intravesical instillation protocol of each patient were in accordance with the “Chinese Diagnosis and Treatment of Urological Diseases Guide” 18. Hydroxycamptothecin, Epirubicin, pyirubicin and gemcitabine were used for perfusion. Besides, the clinical data was obtained by reviewing the medical records of all patients. The nomogram prediction model was validated with an independent cohort of 174 patients from Xiangyang Central Hospital (January 2018 to December 2019).

### Inclusion criteria

Patients were enrolled in this study if they met all the following criteria: (i) newly diagnosed NMIBC; (ii) patients who underwent TURBT and intravesical instillation; (iii) had a complete clinical data record.

### Exclusion criteria

Patients meeting any of the following criteria were excluded: (i) with any a prior history of TURBT or bladder perfusion; (ii) the positive result of urine analysis before instillation; (iii) patients who did not undergo intravesical instillation; (iv) any incomplete clinical data.

### Study methods

All patients had undergone regular perfusion after TURBT. The course of treatment was as as follows: the immediate perfusion therapy was performed within 24 hours after surgery; then once per week for 8 weeks; and then once per month for 12 months. The clinical information of gender, age, diabetes mellitus, hemiplegia, tumor size, tumor location, number of tumors, clinical tumor stage, tumor grade, antibiotic usage, perfusion frequency, perfusion durg, infection, pathogenic bacteria spectrum were collected.

All patients were initially screened with urinary routine tests. Then we collected urine samples (about 10 ml) from patients with “suspicious infections” for urine culture. Collected urine was cultured for bacteria inoculation and then observed morphology of bacterial colony with cultured for 24h at constant 36℃. The colonies were stained by the Gram and counted by microbiological automatic analyzer with identifying. The “infection” was defined as inflammation of urinary tract epithelium caused by bacterial invasion, usually accompanied by bacteriuria and pyuria. In this study, the patients were devided into infection positive group and infection negative group according to urine culture results.

### Statistical analysis

Two-sample t test was used for all continuous measures, and Mann Whitney test was used for grading variables. Chi-square test was used to compare the gender, age, diabetes mellitus, hemiplegia, tumor size, tumor location, number of tumors, clinical tumor stage, tumor grade, antibiotic usage, perfusion frequency, perfusion drug of the two groups. Univariate and multivariate logistic analyses were used to determine the independent risk factors of infection during the intravesical instillation. Based on multivariate logistic analysis, The receiver operating characteristic (ROC) curve and nomogram were generated. The calibration curve was generated to evaluate the consistency between the nomogram-predicted probability with the actual observed probability. SPSS 16.0 and graphpad prism 7 were used for all statistical analyses. R studio 3.5.0 was used to generate nomogram and calibration curve, p value < 0.05 was considered statistical significance.

## Results

### Patient characteristics and pathogenic spectrum analysis

The detailed clinical parameters of enrolled patients in development cohort were presented in Table 1, there was no significant difference in clinical parameters between the two hospitals (all p>0.05). For all bladder cancer patiens with diabetes mellitus, the HbA1c and glucose were controlled within normal range or slight rise. They received standard hypoglycemic therapy with oral anti-hyperglycemia agent and/or insulin subcutaneous injection.

In development cohort, 185 (34.7%) of 533 patients were diagnosed infections. 371 were males and 162 were females, the mean age was 66.3 ± 12.4 years, the median age was 66 years. The mean age of infection negative group was 62.5 ± 11.6 years, the median age was 63 years, and the mean age of infection positive group was 71.1 ± 10.3 years, with a median age of 71 years. Chi-square test showed that age, gender, diabetes mellitus, hemiplegia, tumor size, number of tumors, antibiotic usage and perfusion frequency were significantly different between the two groups (p<0.05), but tumor location, clinical tumor stage, tumor grade, perfusion drug had no statistical difference between two group (Table 2).

In 185 infections, 152 (82.2%) were infected with gram-negative bacteria, 21 (11.4%) were infected with gram-positive bacteria and 12 (6.5%) were fungus. The most common was Escherichia coli with 36.2% among gram-negative bacteria, the second was Acinetobacter baumanii and then Klebsiella Pneumoniae, Pseudomonas aeruginosa and Enterobacter cloacae. The main bacteria were Enterococcus faecalis and Staphylococcus aureus among gram-positive bacteria. Fungus were rare, only 12 cases (Table 3).

### Univariate and multivariate logistic analyses

Univariate logistic analysis showed that tumor size and number of tumors were not the risk factors of infection during the intravesical instillation (p>0.05), whereas age, gender, diabetes mellitus, hemiplegia, antibiotic usage and perfusion frequency were the risk factors (p<0.05). The OR values were as follows: no antibiotics used (OR=3.128), hemiplegia (OR=2.115), perfusion frequency (≥2 times/month, OR=1.646), diabetes mellitus (OR=1.436), age (≥65 years, OR =1.314), female (OR= 1.267) (Table 4).

Moreover, multivariate logistic analysis showed that only diabetes mellitus, hemiplegia, antibiotic usage and perfusion frequency were the independent risk factors of infection during the intravesical instillation (p<0.05), The OR values were as follows: no antibiotics used (OR=3.223), hemiplegia (OR=2.054), perfusion frequency(≥2 times/month, OR=1.819), diabetes mellitus (OR=1.381) (Table 5).

### Construction of nomogram and calibration curve to predict infection during the intravesical instillation

**Figure 1:**
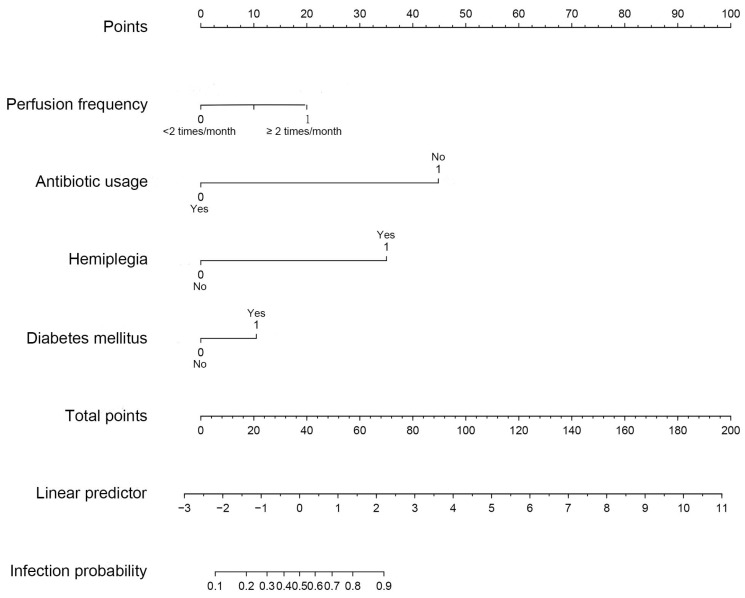
** The nomogram for prediction model of infection during the intravesical instillation.** To estimate the risk of infection, the points for each variable were calculated by drawing a straight line from a patient's variable value to the axis labelled “Points”. The score sum is converted to a probability in the lowest axis.

**Figure 2:**
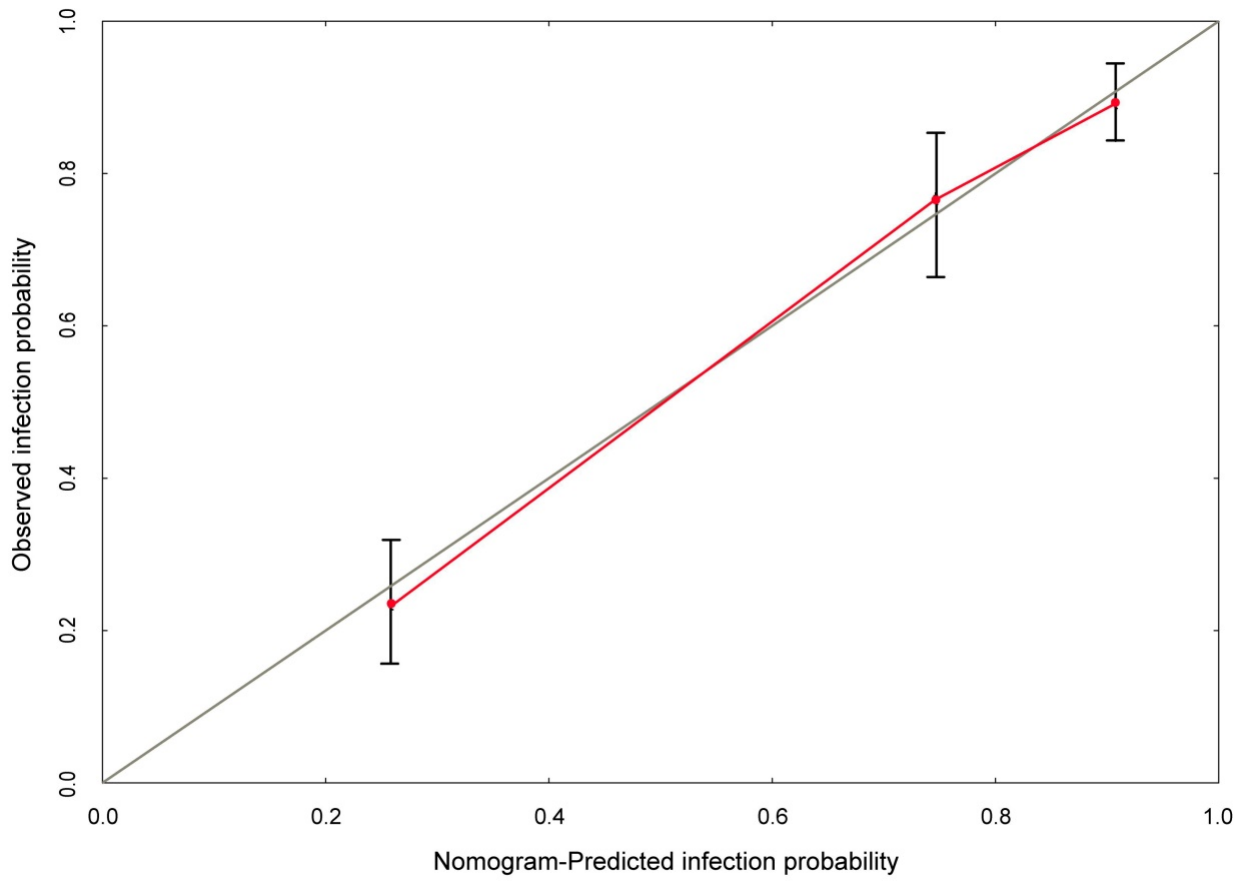
** The calibration curve developed for prediction model of infection during the intravesical instillation.** The nomogram-predicted probability is plotted on the x-axis, and the actual probability is plotted on the y-axis.

Based on multivariate logistic analysis, the factors such as diabetes mellitus, hemiplegia, antibiotic usage and perfusion frequency could be included in the model, and nomogram and calibration curve can be generated to predict infection during the intravesical instillation. For each parameter on the nomogram (Figure 1), calculate the total score to predict the probability of infection for each patient. In the nomogram, we could find the score corresponding to the vertical line of all variable values of the patient on the “score” scale, all variable values scores were accumulated and found the vertical line of the “prediction scale” on the accumulated “total score” scale. According to the score on the prediction scale, the corresponding point was transformed to the corresponding probability on the scale of "infection probability", that was, the possible of infection of patient. The purpose of incorporating the clinical data of each case into nomograph was to carry out matching analysis. The sensitivity and specificity were 82.3% and 78.1%, respectively. The calibration curve (Figure 2) showed that the predicted probability was in good agreement with the actual observed probability of bladder perfusion infection, indicating that the nomogram had great predictive value.

### Evaluation of the prediction model for infection during the intravesical instillation

**Figure 3:**
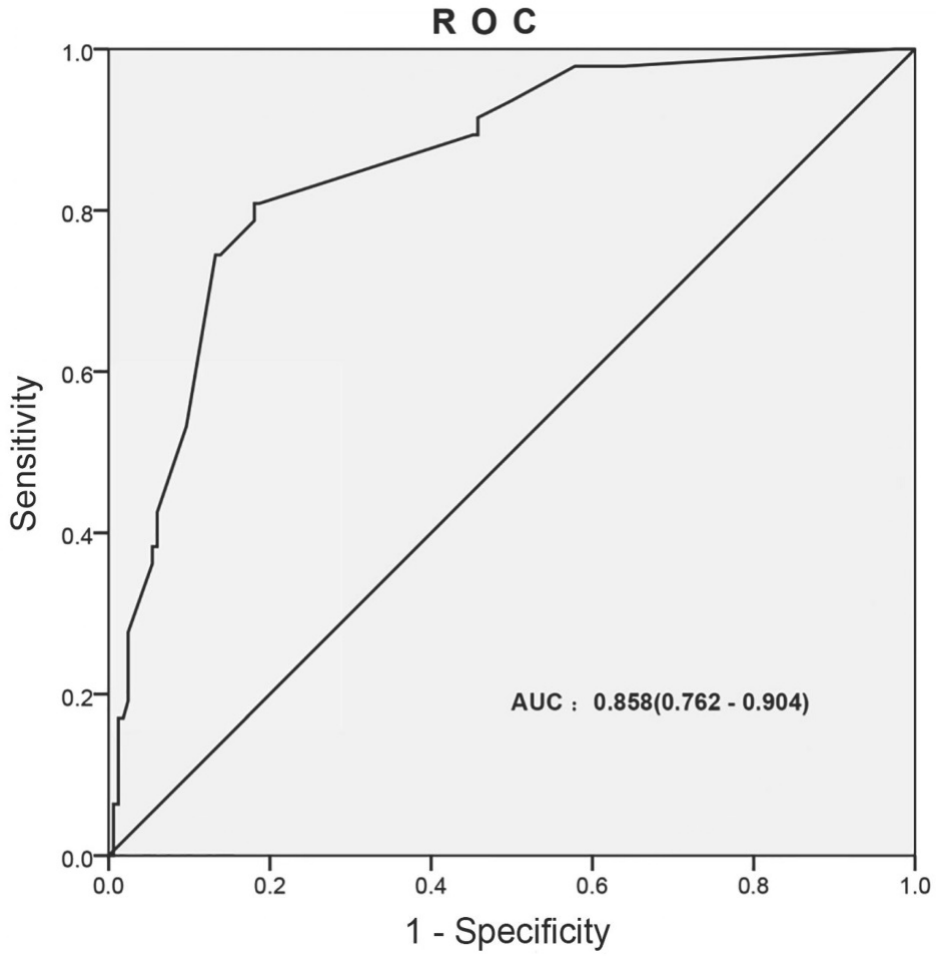
** The ROC curves developed for prediction model of infection during the intravesical instillation.** The AUC of infection prediction model was 0.858 (95%CI: 0.762-0.904).

ROC curve was generated for multivariate logistic analysis to evaluate the value of the prediction model, the “infection occurred” was as the outcome variable (Figure 3). The AUC of prediction model was 0.858 (0.762-0.904). It was been proved again that the prediction model had great value of prediction. To confirm the stability of the model, external data validation was performed, which was independently collected in Xiangyang Central Hospital. The sensitivity was 77.6% and the specificity was 80.4% (Supplementary Table s1).

Taken together, the results showed that the prediction model exhibited high accuracy and stability and was well generalized for other independent datasets.

## Discussion

Previous studies have identified some risk factors for infection of intravesical instillation 19-27. Female patients have special physiological structure, shorter urethra, closed to “contaminated areas” such as the vaginal orifice and anus 19. In the process of intravesical instillation, due to the lack of attention to personal hygiene, inadequate disinfection, hormone physiological changes and invasive operation, ascending infections are highly prone to occur 20-21. Elderly patients with the decline of systemic organs and the body's immune function are also prone to infection. Because of difficulty in urinating, urine retention in the bladder accelerates bacterial growth, long-term indwelling catheterization and hemiplegia, the risk of urinary tract infections often increases 22. The long-term hyperglycemia state reduces the body cell function and anti-infective ability of diabetic patients, what's more, urine glucose becomes a natural medium for the growth of bacteria 23. Frequent intravesical instillation makes the risk of cumulative infection higher 24. Escherichia coli is the most susceptible bacteria in intravesical instillation therapy, not only for the decline in immune function of the patient to the infection which around the urethra, but also the invasive operation to “upward” into the urethra caused infection 25. Proper use of antibiotics can significantly reduce the incidence of infections 26. Some studies also indicated that tumor size and number of tumors may also be factors influencing infection 27.

The univariate logistic analysis showed that age, gender, diabetes mellitus, hemiplegia, antibiotic usage and perfusion frequency were the risk factors of infection during the intravesical instillation, it was the same as previous studies. Furthermore, multivariate logistic analysis showed that only diabetes mellitus, hemiplegia, antibiotic usage and perfusion frequency were the independent risk factors. Based on the multivariate logistic analysis, the prediction nomogram and calibration curve were generated. The nomogram could predict the probability of infection during the intravesical instillation of bladder tumor, with a sensitivity of 82.3% and a specificity of 78.1%. The calibration curve displayed good agreement of the predicted probability with the actual observed probability for infection during the intravesical instillation. In addition, ROC curve was generated for multivariate logistic analysis to evaluate the value of the prediction model, the “infection occurred” was as the outcome variable (Figure 3). The AUC of prediction model was 0.858 (0.762-0.904). External data validation was performed with a sensitivity of 77.6% and a specificity of 80.4%, which indicated that the nomogram had great value of prediction.

Thorough disinfection (operating room, treatment room, perineum parts of patients) before intravesical instillation and aseptic operation (including sterile gloves) during perfusion are very important for infection prevention. It is necessary to assess the possibility of infection according to the patient's age, gender, medical history, perfusion frequency, antibiotic usage, which has great significance for prevention and treatment of the infection during the intravesical instillation.

## Conclusion

The study suggested that the occurrence of the infection during the intravesical instillation came from a variety of factors. Individualized prediction of the probability of infection may reduce the incidence of infection by targeted preventive measures.

## Supplementary Material

Supplementary table.

## Figures and Tables

**Table 1:** Clinical characteristics of enrolled patients in the development cohort.

Variables	Zhongnan Hospital (n=396)	Renmin Hospital (n=137)	*p* value
**Age/years, n (%)**			0.523
Average/Median (Range)	66.1 ± 11.6/66	66.7 ± 10.4/67	
	47 - 86	49 - 86	
**Gender, n (%)**			0.146
Male	272 (68.7)	99 (72.3)	
Female	124 (31.3)	38 (27.7)	
**Diabetes mellitus, n (%)**			0.908
No	312 (78.8)	105 (76.6)	
Yes	84 (21.2)	32 (23.4)	
**Hemiplegia, n (%)**			0.852
No	366 (92.4)	132 (96.4)	
Yes	30 (7.6)	5 (3.6)	
**Tumor size (cm), n (%)**			0.790
<1	259 (65.4)	94 (68.6)	
≥1	137 (34.6)	43 (31.4)	
**Tumor location, n (%)**			0.664
Vesical trigone	110 (27.8)	43 (31.4)	
Sidewall	119 (30.1)	39 (28.5)	
Anterior and posterior wall	125 (31.6)	42 (30.7)	
Others	42 (10.6)	13 (9.5)	
**Number of tumors, n (%)**			0.715
<2	207 (52.3)	68 (49.6)	
≥2	189 (47.7)	69 (50.4)	
**Clinical tumor stage, n (%)**			0.589
Ta -T1	332 (83.8)	123 (89.8)	
T2-T4	64 (16.2)	14 (10.2)	
**Tumor Grade, n (%)**			0.814
G1	193 (48.7)	75 (54.7)	
G2	161 (40.7)	52 (38.0)	
G3	42 (10.6)	10 (7.3)	
**Antibiotic usage, n (%)**			0.167
No	138 (34.8)	35 (25.5)	
Yes	258 (65.2)	102 (74.5)	
**Perfusion frequency, n (%)**			0.392
<2 times/month	177 (44.7)	68 (49.6)	
≥2 times/month	219 (55.3)	69 (50.4)	
**Perfusion durg, n (%)**			0.618
Gemcitabine	214 (54.0)	80 (58.4)	
BCG	48 (12.1)	20 (14.6)	
Hydroxycamptothecin	60 (15.2)	15 (10.9)	
Epirubicin	40 (10.1)	17 (12.4)	
Others	34 (8.6)	5 (3.6)	

**Table 2:** Clinical characteristics of infection during the intravesical instillation of BCa patients.

Variables	All patients (n=533)	Infection negative (n=348)	Infection positive (n=185)	*p* value
**Age/years, n (%)**				0.006
Average/Median (Range)	66.3 ± 12.4/66	62.5 ± 11.6/63	71.1 ± 10.3/71	
	47 - 86	47 - 81	52 - 86	
< 65	143 (26.8)	111(31.9)	32(17.3)	
≥ 65	390 (73.2)	237(68.1)	153(82.7)	
**Gender, n (%)**				0.002
Male	371 (69.6)	296(85.1)	75(40.5)	
Female	162 (30.4)	52(14.9)	110(59.5)	
**Diabetes mellitus, n (%)**				0.011
No	417 (78.2)	291(83.6)	126(68.1)	
Yes	116 (21.8)	57(16.4)	59(31.9)	
**Hemiplegia, n (%)**				0.044
No	498 (93.4)	335(96.3)	163(88.1)	
Yes	35 (6.6)	13(3.7)	22(11.9)	
**Tumor size (cm), n (%)**				0.039
<1	353 (66.2)	252(72.4)	101(54.6)	
≥1	180 (33.8)	96(27.6)	84(45.4)	
**Tumor location, n (%)**				0.250
Vesical trigone	153 (28.7)	92(26.4)	61(33.0)	
Sidewall	158 (29.6)	93(26.7)	65(35.1)	
Anterior and posterior wall	167 (31.3)	121(34.8)	46(24.9)	
Others	55 (10.3)	42(12.1)	13(7.0)	
**Number of tumors, n (%)**				0.042
<2	275 (51.6)	201(57.8)	74(40.0)	
≥2	258 (48.4)	147(42.2)	111(60.0)	
**Clinical tumor stage, n (%)**				0.731
Ta -T1	455 (85.4)	291(83.6)	164(88.6)	
T2-T4	78 (14.6)	57(16.4)	21(11.4)	
**Tumor Grade, n (%)**				0.705
G1	268 (50.3)	169(48.6)	99(53.5)	
G2	213 (40.0)	138(39.7)	75(40.5)	
G3	52 (9.8)	41(11.8)	11(5.9)	
**Antibiotic usage, n (%)**				0.008
No	173 (32.5)	94(27.0)	79(42.7)	
Yes	360 (67.5)	254(73.0)	106(57.3)	
**Perfusion frequency, n (%)**				0.023
<2 times/month	245 (46.0)	185(53.2)	60(32.4)	
≥2 times/month	288 (54.0)	163(46.8)	125(67.6)	
**Perfusion durg, n (%)**				0.846
Gemcitabine	294 (55.2)	193(55.5)	101(54.6)	
BCG	68 (12.8)	41(11.8)	27(14.6)	
Hydroxycamptothecin	75 (14.1)	52(14.9)	23(12.4)	
Epirubicin	57 (10.7)	38(10.9)	19(10.3)	
Others	39 (7.3)	24(6.9)	15(8.1)	

**Table 3:** Pathogenic spectrum of patients with positive infection.

Pathogenic bacteria	Cases	%
**Gram-negative bacteria**	**152**	**82.2**
*Escherichia coli*	67	36.2
*Klebsiella pneumoniae*	20	10.8
*Acinetobacter baumannii*	28	15.1
*Pseudomonas aeruginosa*	25	13.5
*Enterobacter cloacae*	12	6.5
**Gram-positive bacteria**	**21**	**11.4**
*Enterococcus faecalis*	15	8.1
*Staphylococcus aureus*	6	3.2
**Fungus**	**12**	**6.5**
*Candida albicans*	12	6.5
Total	185	100%

**Table 4:** Univariate logistic analysis for infection during the intravesical instillation of BCa patients.

Variables	OR	95% CI	*p* value
**Age/years** (<65 vs. ≥ 65)	1.314	1.119 - 1.622	0.042
**Gender** (male/female)	1.267	1.107 - 1.381	0.036
**Diabetes mellitus** (no/yes)	1.436	1.246 - 1.855	0.008
**Hemiplegia** (no/yes)	2.115	1.967 - 2.662	0.002
**Tumor size/cm** (<1 vs. ≥ 1)	1.021	0.862 - 1.136	0.145
**Number of tumors** (<2 vs. ≥ 2)	1.132	0.934 - 1.219	0.103
**Antibiotic usage** (no/yes)	3.128	2.425 - 4.018	< 0.001
**Perfusion frequency** (<2 times/month vs. ≥ 2 times/month)	1.646	1.328 - 1.983	0.004

**Table 5:** Multivariate logistic analysis for infection during the intravesical instillation of BCa patients.

Variables	OR	95% CI	*p* value
**Diabetes mellitus** (no/yes)	1.381	1.181 - 1.685	0.009
**Hemiplegia** (no/yes)	2.054	1.653 - 2.546	0.011
**Antibiotic usage** (no/yes)	3.223	2.520 - 4.148	< 0.001
**Perfusion frequency** (<2 times/month vs. ≥ 2 times/month)	1.819	1.412 - 2.037	0.003

## Abbreviations

AUC: Areas Under the Curves
BCa: Bladder Cancer
MIBC: Muscle Invasive Bladder cancer
NMIBC: Non-Muscle Invasive Bladder Cancer
ROC: Receiver Operating Characteristic
TURBT: Transurethral Resection of Bladder Tumor

## References

[1] Ferlay J, Soerjomataram I, Dikshit R (2015). Cancer incidence and mortality worldwide: sources, methods and major patterns in GLOBOCAN 2012. Int J Cancer.

[2] Garg M (2015). Urothelial cancer stem cells and epithelial plasticity: current concepts and therapeutic implications in bladder cancer. Cancer Metastasis Rev.

[3] Miller KD, Siegel RL, Lin CC (2016). Cancer treatment and survivorship statistics, 2016. CA Cancer J Clin.

[4] Kramer MW, Altieri V, Hurle R (2017). Current Evidence of Transurethral En-bloc Resection of Nonmuscle Invasive Bladder Cancer. Eur Urol Focus.

[5] Kramer MW, Altieri V, Hurle R (2017). Current Evidence of Transurethral En-bloc Resection of Nonmuscle Invasive Bladder Cancer. Eur Urol Focus.

[6] Feifer A, Xie X, Brophy JM (2010). Contemporary cost analysis of single instillation of mitomycin after transurethral resection of bladder tumor in a universal health care system. Urology.

[7] Kumari K, Pradeep I, Kakkar A (2019). BK polyomavirus and urothelial carcinoma: Experience at a tertiary care centre in India with review of literature. Ann Diagn Pathol.

[8] Kohada Y, Goriki A, Yukihiro K, Ohara S, Kajiwara M (2019). The risk factors of urinary tract infection after transurethral resection of bladder tumors. World J Urol.

[9] Babjuk M, Burger M, Compérat EM (2019). European Association of Urology Guidelines on Non-muscle-invasive Bladder Cancer (TaT1 and Carcinoma In Situ) - 2019 Update. Eur Urol.

[10] Pereira JF, Pareek G, Mueller-Leonhard C (2019). The Perioperative Morbidity of Transurethral Resection of Bladder Tumor: Implications for Quality Improvement. Urology.

[11] Bolat D, Gunlusoy B, Aydogdu O (2018). Comparing the short - term outcomes and complications of monopolar and bipolar transurethral resection of bladder tumors in patients with coronary artery disese: a prospective, randomized, controlled study. Int Braz J Urol.

[12] Kim BS, Tae BS, Ku JH (2018). Rate and association of lower urinary tract infection with recurrence after transurethral resection of bladder tumor. Investig Clin Urol.

[13] Bolat D, Gunlusoy B, Degirmenci T (2016). Comparing the short-term outcomes and complications of monopolar and bipolar transurethral resection of non-muscle invasive bladder cancers: a prospective, randomized, controlled study. Arch Esp Urol.

[14] Doyle E, Crew J, Mostafid H (2019). Urothelial cancer: a narrative review of the role of novel immunotherapeutic agents with particular reference to the management of non-muscle-invasive disease. BJU Int.

[15] Koo K, Zubkoff L, Sirovich BE (2017). The Burden of Cystoscopic Bladder Cancer Surveillance: Anxiety, Discomfort, and Patient Preferences for Decision Making. Urology.

[16] Domingos-Pereira S, Sathiyanadan K, La Rosa S (2019). Intravesical Ty21a Vaccine Promotes Dendritic Cells and T Cell-Mediated Tumor Regression in the MB49 Bladder Cancer Model. Cancer Immunol Res.

[17] Sousa A, Inman BA, Piñeiro I (2014). A clinical trial of neoadjuvant hyperthermic intravesical chemotherapy (HIVEC) for treating intermediate and high-risk non-muscle invasive bladder cancer. Int J Hyperthermia.

[18] Zhang J, Wang Y, Weng H (2019). Management of non-muscle-invasive bladder cancer: quality of clinical practice guidelines and variations in recommendations. BMC Cancer.

[19] Madero-Morales PA, Robles-Torres JI, Vizcarra-Mata G (2019). Randomized Clinical Trial Using Sterile Single Use and Reused Polyvinylchloride Catheters for Intermittent Catheterization with a Clean Technique in Spina Bifida Cases: Short-Term Urinary Tract Infection Outcomes. J Urol.

[20] Malde S, Spilotros M, Wilson A (2017). The uses and outcomes of the Martius fat pad in female urology. World J Urol.

[21] Chung E, Tse V, Chan L (2010). Mid-urethral synthetic slings in the treatment of urodynamic female stress urinary incontinence without concomitant pelvic prolapse repair: 4-year health-related quality of life outcomes. BJU Int.

[22] Petty LA, Vaughn VM, Flanders SA (2019). Risk Factors and Outcomes Associated With Treatment of Asymptomatic Bacteriuria in Hospitalized Patients. JAMA Intern Med.

[23] Fünfstück R, Nicolle LE, Hanefeld M, Naber KG (2012). Urinary tract infection in patients with diabetes mellitus. Clin Nephrol.

[24] Gregg JR, McCormick B, Wang L (2016). Short term complications from transurethral resection of bladder tumor. Can J Urol.

[25] Matulewicz RS, Sharma V, McGuire BB (2015). The effect of surgical duration of transurethral resection of bladder tumors on postoperative complications: An analysis of ACS NSQIP data. Urol Oncol.

[26] Kowalik C, Gee JR, Sorcini A (2014). Underutilization of immediate intravesical chemotherapy following TURBT: results from NSQIP. Can J Urol.

[27] Murakami M, Kiyota H, Kasai K Antimicrobial prophylaxis for transurethral resection of bladder tumor: A retrospective comparison of preoperative single-dose administration of piperacillin and tazobactam/piperacillin. 2018; 24: 954-957.

