# Supplementary material for: Establishing a prediction model of infection during the intravesical instillation of bladder cancer: a multicenter retrospective study
Source: J Cancer. 2020 Apr 27;11(15):4324–31. doi: 10.7150/jca.45055 (PMC7255357; doi:10.7150/jca.45055)
Supplement: Supplementary file 1 — Supplementary table. [file jcav11p4324s1.pdf]

Supplementary Table s1. External data validation of the prediction model for infection during the intravesical instillation.

		**Infection (Validation cohort)**		**Total**
		No	Yes	
**Infection (Prediction model)**	No	86	21	107
	Yes	15	52	67
**Total**		101	73	174

Sensitivity =52/(52+15)=77.6%

Specificity =86/(86+21)=80.4%
